# Feasibility, acceptability and sustainability of family-led postnatal care model: a multisite mixed study in Ada’a District, Ethiopia

**DOI:** 10.1136/bmjph-2025-003548

**Published:** 2026-05-12

**Authors:** Gadise Bekele Regassa, Konjit Wolde, Dedefo Teno Teshite, Lello Amdissa, Walelegn Worku Yallew, Solomon Getachew Alem, Pooja Sripad, Anne Hyre, Stephanie Suhowatsky, Lisa Noguchi, Alemayehu Worku Yalew, Della Berhanu

**Affiliations:** 1 Public Health, Addis Continental Institute of Public Health, Addis Ababa, Ethiopia; 2 Jhpiego Ethiopia, Addis Ababa, Ethiopia; 3 Jhpiego, Baltimore, Maryland, USA; 4 Epidemiology, JHSPH, Baltimore, Maryland, USA; 5 Department of Epidemiology and Biostatistics, Addis Ababa University School of Public Health, Addis Ababa, Ethiopia; 6 Department of Disease Control, London School of Hygiene and Tropical Medicine Faculty of Infectious and Tropical Diseases, London, UK; 7 Ethiopian Public Health Institute, Addis Ababa, Ethiopia

**Keywords:** Public Health, Qualitative Research, Community Participation

## Abstract

**Introduction:**

Family-led postnatal care is a self-care innovation designed to support mothers and newborns during the first week after birth. In the model, midwives invited family members to attend discharge counselling and demonstration on how to assess the mother and newborn using pictorial checklist. Families were provided with the checklist and guidance on retrieving a home care kit containing a blood pressure monitor, thermometer and health-education booklet from a volunteer community custodian. Families used the checklist and kit at home to assess the health of the mother and newborn for 6 days and then returned the completed checklist and kit to the custodian. This study aimed to assess the feasibility, acceptability and sustainability of the family-led postnatal care model.

**Methods:**

An explanatory sequential mixed-method design was conducted in post-intervention between February and April 2023 in four health centres and their catchment areas in Ada’a District, Oromia, Ethiopia. The study drew on a cross-sectional survey, review of facility registers and implementation checklists, and qualitative interviews. The quantitative component included 110 women who had recently given birth. The qualitative component included interviews with postpartum women, husbands/partners, family members, midwives/nurses, health extension workers, home care kit custodians and health managers (n=88). Descriptive analysis using frequency and proportion was computed. Qualitative data were analysed using reflexive thematic analysis.

**Results:**

Participants at facility and community levels felt that family-led postnatal care was feasible and acceptable due to the easy-to-use materials for varied literacy levels, its influence on spouses/partners and families to support mothers, and its empowerment of women to recognise signs that require care-seeking. All health centres continued the family-led postnatal care model, and 97% of kits were functional 6 months after the end of project support.

**Conclusions:**

Our results indicate that family-led postnatal care is a promising approach that can be tested in other settings.

**Trial registration number:**

NCT05563974.

**WHAT IS ALREADY KNOWN ON THIS TOPIC**

- In Ethiopia, 80% of mothers and newborns receive no postnatal care.
- Innovative models are needed to deliver postnatal care for mothers and newborns in Ethiopia.

**WHAT THIS STUDY ADDS**

- This family-led postnatal care model’s culturally sensitive, low-literacy tools and integration into the healthcare system have demonstrated significant feasibility, acceptability and early signs of sustainability for a self-care approach to postnatal care in Ethiopia’s rural, resource-limited settings.

**HOW THIS STUDY MIGHT AFFECT RESEARCH, PRACTICE OR POLICY**

- Family-led postnatal care, a self-care model for postnatal care, is a promising approach to test in other settings.

## Background

 In Ethiopia, maternal and newborn mortality are high at 412 deaths per 100 000 live births and 33 deaths per 1000 live births, respectively.^1^ These figures are significantly higher in rural areas where access to healthcare services is limited. The postnatal period, which encompasses the first 6 weeks after childbirth, is a critical time for both mother and newborn, accounting for up to 50% of maternal deaths and 40% of newborn deaths.^2^ Despite this, only 17% of Ethiopian women receive postnatal care (PNC) within the recommended 48 hours after birth. This is largely due to cultural barriers that prevent women from leaving the house in the first weeks after birth, lack of knowledge of the importance of PNC, perceived low quality of services, shortages in healthcare professionals and inadequate infrastructure, especially in rural and resource-limited settings.^13^,^6^ Consequently, innovative care models are urgently needed to improve PNC access and health outcomes.

The WHO emphasises the importance of locally tailored interventions, such as home-based postnatal visits, to monitor maternal and newborn health and enhance early detection of complications to initiate prompt seeking of care at the nearest health facility.^7^ Different studies highlight that a combination of social support groups, community mobilisation and health worker training is crucial in overcoming cultural and systemic barriers to accessing PNC. These findings suggest that when community-driven innovations are integrated into PNC programmes, maternal and newborn mortality can be reduced significantly in low-resource settings.^8^,^10^

Self-care practices have also emerged as a critical component in empowering mothers to manage their own health and that of their newborns during the postnatal period. The 2019 ‘WHO guideline on self-care interventions for health’ outlines essential self-care practices such as exclusive breastfeeding, proper hygiene, nutritional management and self-monitoring of postnatal symptoms.^11^ In a study conducted in southwest Ethiopia, self-care practices were found to be inconsistent, with adherence rates as low as 30% for wound care and hygiene management due to socioeconomic challenges, limited healthcare access and cultural barriers.^12^ Evidence suggests that family involvement in PNC can also reduce newborn mortality and increase maternal satisfaction with care.^13 14^ These findings emphasise the need for more research to explore the role of self-care interventions in rural Ethiopian communities, particularly in overcoming the socio-economic and cultural barriers that limit access to PNC.

This study aimed to explore the feasibility, acceptability, effectiveness and sustainability of a family-led PNC (FPNC) model, an innovative model for PNC for mothers and newborns in Ethiopia. Effectiveness of the FPNC model in increasing PNC coverage has been reported separately.^15^ This paper focuses on the feasibility and acceptability of the family-led model, which are critical in determining its sustainability and scalability. It emphasises the importance of evaluating the practicality of implementing such models within existing healthcare systems and community structures as essential.^16^ Acceptability studies play a key role in ensuring that health interventions are culturally appropriate and well-received by the target population.^17^ This study addressed these factors and aimed to provide insights that can inform the broader implementation of FPNC models in settings facing similar challenges.

## Methods

### Study design

The study applied an explanatory sequential mixed-method design conducted postintervention between February to April 2023.^18^ Quantitative data were collected first through a cross-sectional survey, review of registers and checklists. These findings informed the subsequent qualitative phase, which comprised in-depth interviews and key-informant interviews.

### Study setting

This study took place in four health centres and their catchment areas in Ada’a District, Oromia Region, Ethiopia. Ada’a is one of the districts in East Showa zone, where most of the population resides in rural areas. In Ethiopia, districts’ primary healthcare system consists of a primary hospital, five health centres and five health posts under each health centre. Health extension workers (HEWs) provide preventive and curative services, including maternal, newborn and child health services, at health posts and through home visits. Deliveries are expected to take place in health centres and hospitals.

Four health centres in Ada’a District were purposively selected to participate, and all surrounding communities were eligible to participate. One health centre was involved in the conceptualisation, design and beta testing of the FPNC model components and was excluded from the study.

### Description of the FPNC intervention

Human-centred design activities revealed key barriers to timely PNC use in the local context that informed intervention design. Mothers and newborns were expected to stay home during the first 42 days, particularly during the first 10 days after birth. Mothers relied mainly on their husbands/partners, immediate family, religious leaders and neighbours for advice and decision-making. They often felt pressured to ‘prove’ themselves to be capable mothers and feared being inadequate at caring for their newborn or recognising problems. Health facilities were typically seen as a last resort, sought only in critical situations.^19^

**Figure 1:**
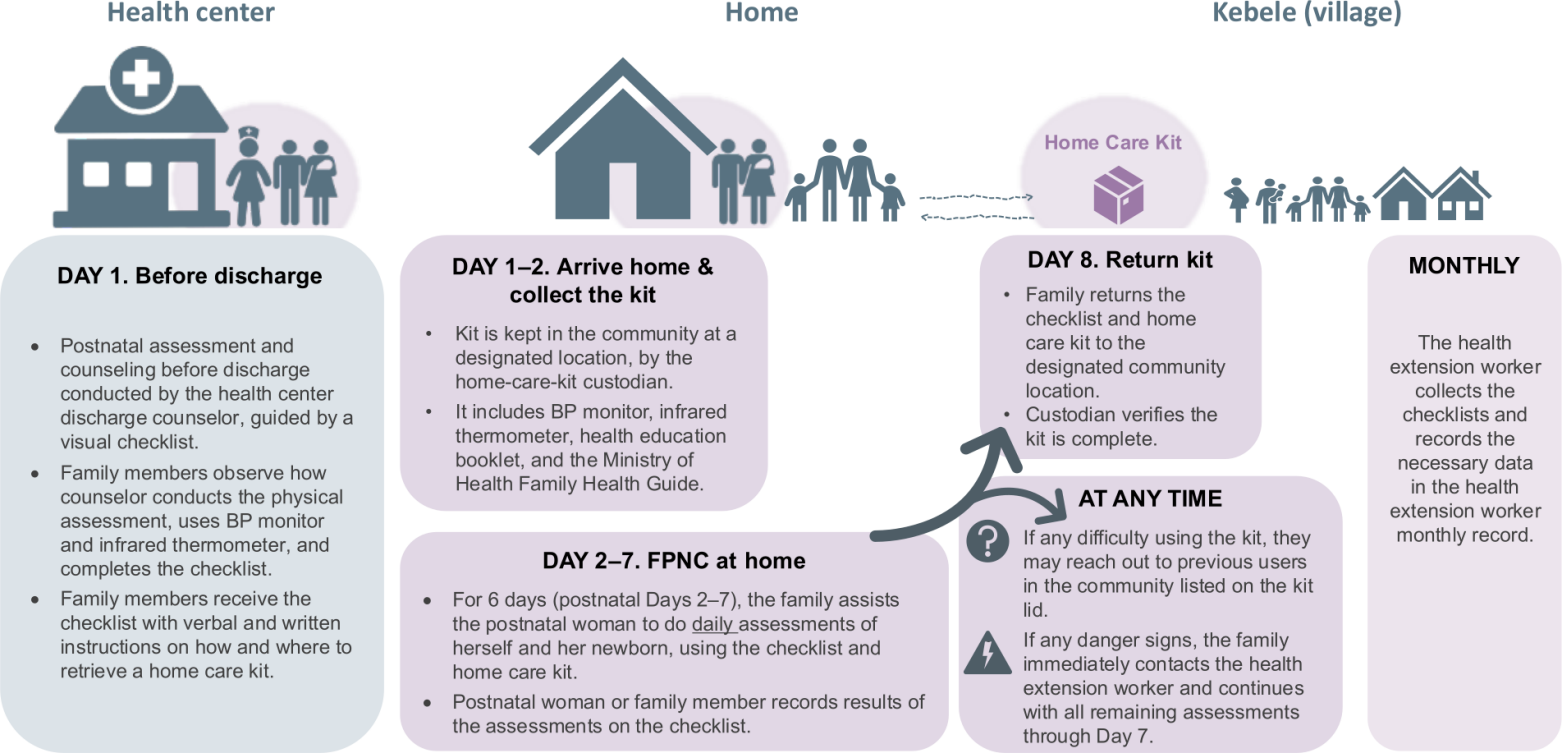
Implementation of the FPNC intervention. BP, blood pressure; FPNC, family-led postnatal care.

These insights informed the FPNC intervention, a novel model for reaching women who have given birth and newborns during the first week after birth with key PNC services, founded on self-care principles. FPNC consists of the components described in figure 1.

The intervention included training midwives, nurses and health officers to equip them with skills to provide predischarge care effectively as part of FPNC. The study team developed a script for the healthcare providers to use alongside a checklist during discharge, ensuring consistent communication and assessments. The checklist included manual assessments of mothers and newborns and assessments using devices such as a thermometer and blood pressure (BP) monitor, which were made available to families in the community as a home-care kit (HCK).

The study team also developed a photo booklet, which was included in the HCK, that featured images of normal and abnormal signs in mothers and newborns to aid caregivers in identifying potential issues. The HCK itself was a portable case, labelled with a sticker containing contact information for previous users, allowing current users to seek guidance if needed.

The study team, along with the HEW and community leaders, designated custodians to manage the kits, based on specific criteria. The custodians maintained a register to record who borrowed the HCK and returned it with the checklist. They also inspected the kits on return, recharging any components as necessary.

### Patient and public involvement

We collaborated with one of the health centres to develop the checklist and identify the necessary equipment for the homecare kit and pilot it. In partnership with HEWs and community leaders, we identified community custodians. Additionally, we worked closely with these custodians to establish a mechanism for tracking and managing the homecare kits.

We engaged the district office to introduce the intervention, health centres to oversee the distribution of homecare kits, and HEWs to manage data on PNC coverage by collecting the checklist provided to mothers. Finally, we invited policy makers, participants and community members to a results dissemination meeting and feedback was provided.

### Sample size

#### Quantitative sample

The sample size was calculated using the 2016 Ethiopian Demographic and Health Survey coverage of PNC within 24 hours of 17%.(1) With a desired increase to 45% due to the intervention, a 5% level of significance, 80% power, a design effect of 2.0, and a non-response rate of 10%, the minimum sample size required to measure our primary outcome was 109 women who had given birth at both preintervention and postintervention.^1^ This paper focuses on the postintervention survey only. The quantitative sample included women who gave birth at the four study health centres; women were included sequentially until the sample size was met.

#### Qualitative sample

40 key-informant interviews and 48 in-depth interviews were conducted. The qualitative sample included: (1) 19 eligible women who gave birth at the four study health centres; (2) 15 eligible partners; (3) 14 eligible women’s family members; (4) nine discharge counsellors at the four health centres; (5) 15 HEWs in the catchment area of the four health centres; (6) eight HCK custodians in the community and (7) eight district health managers and health centre managers. All participants were a minimum of 15 years old. Purposive sampling was employed, based on the completed postnatal checklists and consultation with HEWs and the HCK custodians (table 1). Data collection for the qualitative component continued until thematic saturation was reached, ensuring that no new insights emerged from additional interviews.

**Table 1:** Inclusion criteria of the study participants for qualitative data collection

Study population	Description
Health centre discharge counsellors	All eligible discharge counsellors, staff at intervention health centre who were midwives, nurses and/or health officers
Women who have given birth	Selected eligible women who gave birth at intervention health centres (women who completed all postnatal checks, women who missed a postnatal check, women who identified postnatal danger signs and sought care from a health provider, women who identified postnatal danger signs, but who did not seek care)
Family members	Family members of eligible women present at discharge
Husbands/partners	Husbands/partners of eligible women
Health extension workers	All health extension workers working in catchment area of intervention health centres
Home care kit custodians	Community leaders serving as home care kit custodians in catchment area of intervention health centres
Health managers	Health managers overseeing maternal/newborn health activities in Ada’a District; heads of intervention health centres

### Data collection procedure

#### Quantitative

Training sessions were conducted for field data collectors and supervisors, focusing on study objectives, procedures and ethical considerations. A comprehensive survey manual of standard operating procedures was developed and used during the training, pilot phase and survey execution. The training also covered the appropriate use of tablets for data entry during data collection.

Health centre staff responsible for postnatal discharge at the four selected centres were tasked with screening mothers for study eligibility. Before discharge, eligible mothers were asked if they were interested in learning more about the study. If they expressed interest, they were referred to FPNC study staff. Study personnel on-site approached the mothers and their family members, provided further details about the study, addressed any questions and obtained oral informed consent. For those who consented, contact information was collected and passed on to the local study enumerators. Designated data collectors visited the mothers’ homes on postnatal Day 8 to conduct surveys (online supplemental questionnaire).

#### Qualitative

HEWs and HCK custodians were invited to participate in community orientations. After the postintervention quantitative survey, research assistants approached health centre discharge counsellors; women who have given birth and family members, husbands/partners; HEWs; and HCK custodians at a convenient place, explained the study and obtained oral informed consent before they participated in the qualitative data collection (online supplemental interview guides).

In-depth interviews were conducted at the home of women who gave birth at intervention facilities, according to the inclusion criteria outlined in table 1. Key-informant interviews were conducted with the discharge counsellors, HEWs, health managers and HCK custodians who were involved in FPNC intervention implementation. The interviews were conducted at their place of work. Each interview was audio recorded with the permission of the participants to ensure accurate data capture. Local language Afaan Oromo and Amharic was used for both quantitative and qualitative data collection.

### Outcome measures

Women who gave birth in study facilities were interviewed using a structured questionnaire to collect information on basic sociodemographic data (age, educational status and marital status). In addition, the following information on the primary and secondary implementation outcomes was collected from the study participants.^20^

#### Feasibility

- Feasibility of FPNC at health centre, community and at household level.
- Proportion of women discharged from the facility whose family members retrieved the HCK; proportion of women who report successful use of BP cuff; proportion of women who report successful use of thermometer; proportion of HCKs returned by day 8; proportion of checklists returned with the HCK; and proportion of checklists collected by HEW.

#### Acceptability

- Proportion of women who give birth at health facility and accept a checklist from the discharge counsellor; proportion of women who report they prefer this PNC approach; and proportion of women who report confidence in this PNC approach.
- Perceptions of HCK custodians, health managers, midwives/nurses and HEWs regarding the FPNC approach.
- Perceptions of a woman’s partner and family regarding the FPNC approach.

#### Sustainability

Continued implementation of the FPNC model at the health centres and communities 6 months after the postintervention survey was completed.

### Data management and analysis

Quantitative data were collected electronically using SurveyCTO application on tablets. Data from the field were synchronised daily to the central cloud server. Study team leads conducted daily checks on completeness and consistency of the collected data. The electronic data capturing system had built-in logic, range and skip patterns to limit data inaccuracies. Data were cleaned before the main analysis. Descriptive analysis using frequency and proportion was computed. STATA V.15 software was used to analyse the data.

Qualitative data were collected after the quantitative survey and were used to contextualise the quantitative findings. Qualitative data were interpreted using reflexive thematic analysis to understand participants’ experiences with FPNC and thoughts on its feasibility, acceptability and sustainability.^21^ The study team actively constructed themes and derived patterns (or meanings) collectively. Inductive coding was used. Themes were generated by the study team familiarising themselves with the data, generating initial codes, searching for themes, reviewing themes and defining and naming themes through a deliberative process. Data were transcribed and translated to English. Open code software was used for analysing the qualitative data.^22^

Quantitative and qualitative findings are presented separately in the results section. Integration and interpretation is incorporated in the discussion section. The data supporting the findings of this study are available on the Figshare repository.^23^

## Results

### Characteristics of women who have given birth

More than 90% of the women who have given birth in the postintervention survey were 18 to 35 years old. Three-quarters had no education or a primary education, and 24% had secondary or above. Orthodox religion was the predominant religion (87%). More than 90% of interviewed women were either currently married or cohabiting (table 2).

**Table 2:** Background characteristics of women included in post-intervention surveys (February–April 2023), Ada’a District, Ethiopia

	N=110
	n (%)
Age in years	
15–24	45 (41)
25–34	49 (45)
≥35	16 (14)
Education level	
None	26 (24)
Primary	58 (52)
Secondary and above	26 (24)
Religion	
Orthodox	96 (87)
Other	14 (13)
Marital status	
Currently married/cohabiting	103 (94)
Other	7 (6)

### Feasibility of FPNC approach

The feasibility of the FPNC model from interviewing mothers and reviewing registers is shown in table 3. At health centres, of the 115 women who gave birth during the endline survey, 96% were offered a checklist and informed about HCK.

**Table 3:** Feasibility of the family-led postnatal care approach, reported in postintervention surveys (February–April 2023), Ada’a District, Ethiopia

	N=110
	n (%)
Feasibility	
Challenges with obtaining the Home Care Kit (HCK)	
Had no challenges in receiving the HCK from the custodian	99 (90)
The HCK custodians were not available	4 (4)
There were no HCKs available	3 (3)
I didn’t have a family member available to retrieve the HCK	3 (3)
No challenges in returning the HCK to the custodian	102 (93)
Data extracted from HCK register	N=99*
HCKs returned	99 (100)
Checklists returned with the HCKs	97 (98)
Checklists collected by health extension worker	73 (74)
Challenges while using the HCK at home	
I did not face any challenges	99 (90)
The blood pressure apparatus became non-functional	2 (2)
The thermometer became non-functional	2 (2)
Used the photo health education booklet	42 (38)
Among those who used the booklet, no challenges were encountered	41 (98)

* Data are for 99 women registered on the HCK register.

90% of the women reported no difficulties in receiving and returning the HCK to the custodian. Among those who reported a challenge, the most common challenges were that the HCK custodian was not available, there were no HCKs available, and the postnatal woman did not have a family member available to retrieve the HCK. Data abstracted from the HCK registers shows that 100% of the HCK and 98% of the checklist were returned with the HCKs (table 3).

**Figure 2:**
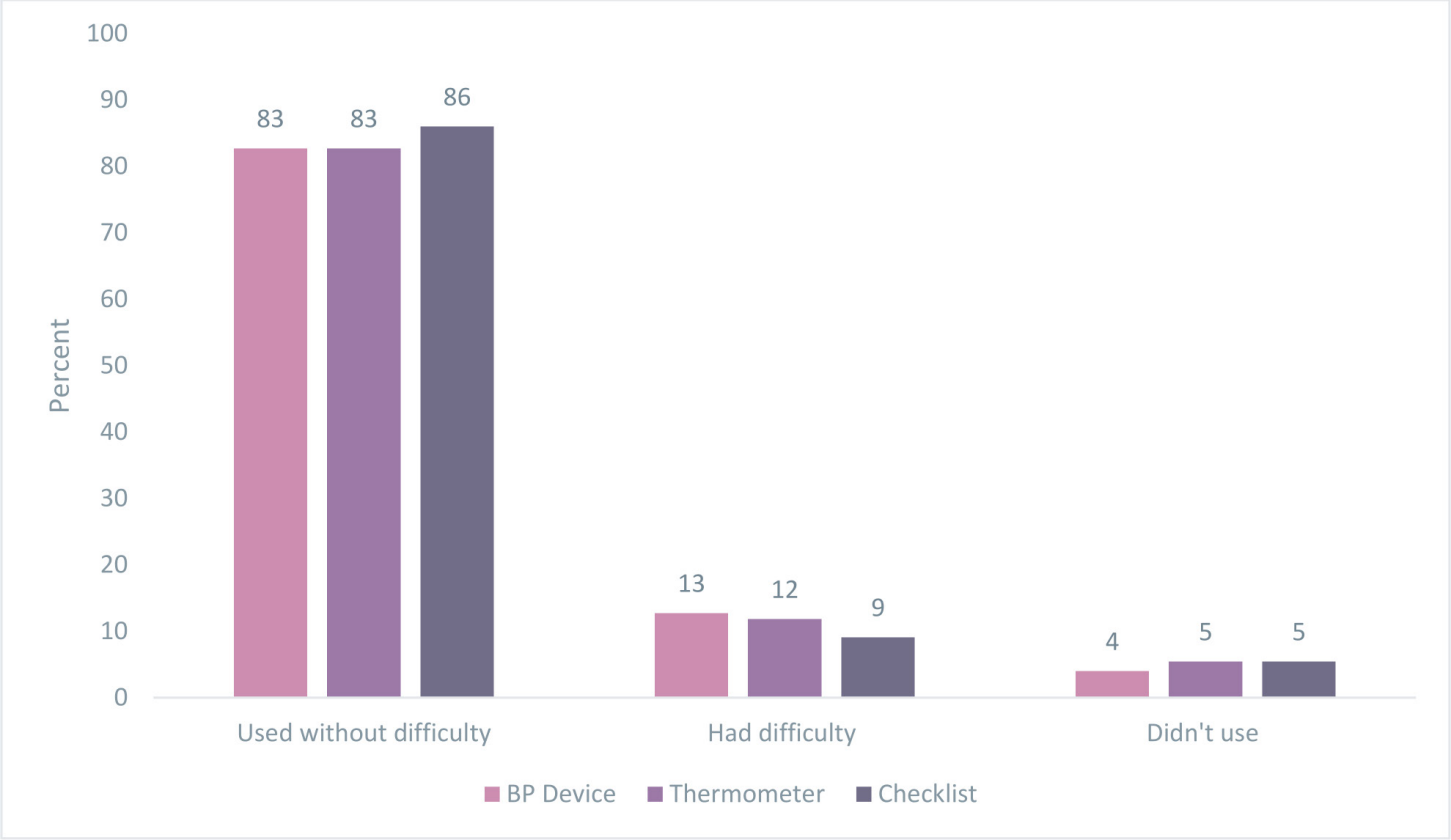
Feasibility of women who have given birth and their families using the BP monitor, thermometer and checklist during the postintervention period (February–April 2023) N=110, Ada’a District, Ethiopia. BP, blood pressure.

Most women did not face challenges while using the HCK. When there was a problem, non-functioning BP monitors and thermometers were the most common challenges encountered. In contrast, only a few women (38%) used the photo health-education booklet. Among those who used it, nearly all reported not encountering any challenge in its use (table 3). More than 80% of the women used the BP device, thermometer and checklist without difficulty, and only less than 14% of women had difficulty (figure 2). About 73% of the returned checklists were collected by the HEW (table 3).

### Acceptability of FPNC approach

Out of 110 women who were discharged with a checklist, 105 (96%) retrieved the HCK from the custodians in their community. Most of the women (95%) preferred the FPNC approach compared with the traditional PNC by a healthcare provider during a previous childbirth experience. 95% of the women felt confident that they, along with their babies, had received good quality PNC when using the HCK at home. Almost all women wanted to use the FPNC approach for future births. Women reported that husbands/partners were ‘very involved’ in the FPNC model (table 4).

**Table 4:** Acceptability of the family-led postnatal care (FPNC) approach, reported in postintervention surveys (February–April 2023), Ada’a District, Ethiopia

	N=110
	n (%)
Acceptability	
Obtained the HCK after discharge	105 (96)
PNC approach preference compared with previous birth	
Prefer traditional PNC by a healthcare provider	2 (2)
Prefer FPNC	104 (95)
No previous PNC or comparison	4 (3)
Confident in the quality of PNC when using HCK	104 (95)
Level of confidence in the quality of assessments using the HCK	
Highly confident	98 (89)
Moderately confident	11 (10)
Not sure or do not know	1 (1)
PNC preference for future birth	
FPNC home-based approach	107 (97)
PNC by a health extension worker or at health centre	3 (3)
Husband or partner involvement in the FPNC	
Very involved	96 (87)
Somewhat involved	8 (7)
Not involved	6 (6)

HCK, Home Care Kit; PNC, postnatal care.

**Figure 3:**
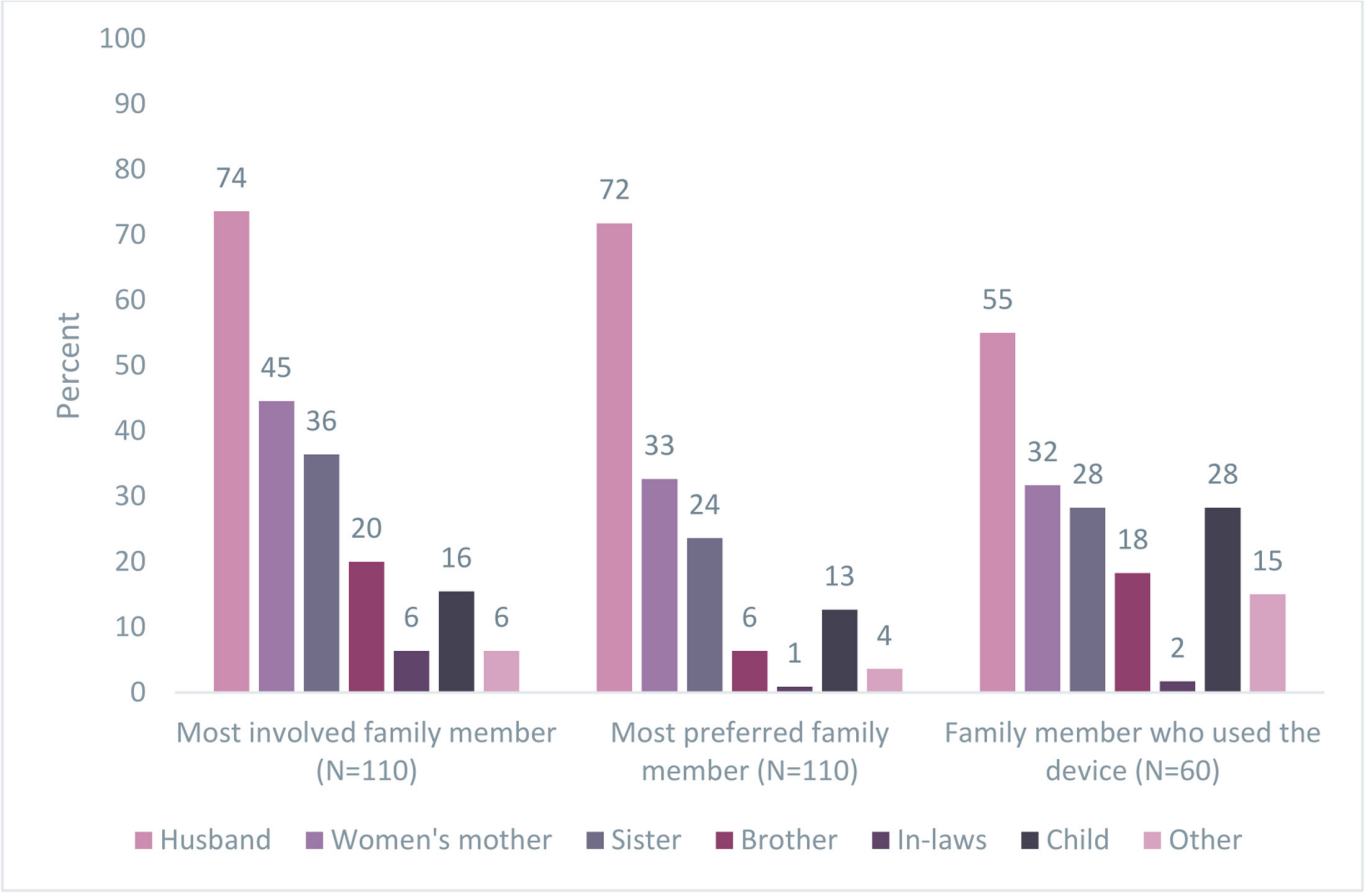
Family involvement and use of devices in the postintervention period (February–April 2023), Ada’a District, Ethiopia.

Husbands or partners were the most involved family members in the FPNC approach and their preferred roles in FPNC were most corroborated by women. Husbands were the most preferred, followed by the women who have given birth’s mothers, to use the devices to perform health checks (figure 3). In addition, older children, mothers-in-law and sisters provided checks to mothers and newborns.

### Sustainability of FPNC approach

All four health centres were still providing FPNC services 6 months after the postintervention survey was completed, as reported in October 2023. Among 64 HCKs provided to custodians, 62 (97%) were functional.

### Qualitative finding

#### Feasibility

The major themes that emerged from analysis of the key-informant and in-depth interviews under feasibility included the feasibility of implementing the FPNC model at the health centre, community and household levels (online supplemental table 2).

#### Feasibility: at health centre

Most discharge counsellors managed to follow the scripts during discharge counselling, although they stated that the counselling focused more on how to use the BP and thermometer devices than on other danger signs. Regarding the adequacy of the information and checks for detecting danger signs in the mother and newborn, they all agreed that the materials were enough and were also feasible for individuals with minimal or no education. However, some raised the concern that there was only one midwife covering antenatal care, birth and PNC, which resulted in increased workloads when doing the discharge counselling. They also stated that it took providers more time to counsel families with minimal or no education.

Health managers agreed that the FPNC approach had made the postnatal discharge systematic and effective because of the script and checklist provided. A health manager at a woreda stated:

In the past, the healthcare provider might have missed important details while providing the service because they lacked an organized checklist form, but now that FPNC has provided the checklist, we follow it, which helps the healthcare provider to provide discharge-counselling services at the required level. HM_AdaHO_KII_01

The discharge counselling also included family members. As such, women who have given birth and family members were happy to have family members present during counselling. A mother explains:

Very good, being with the family first makes my family understand it too, because that advice is very important to me and the baby, and my family can do the same help for me and the baby that the health worker did for us. It’s very good for me. Mother_Denkaka_IDI_02

A husband commented on the discharge counselling:

It’s good because knowing what’s going to happen beforehand means there’s nothing to panic about. Because if something happens, they said come to us [the health centre]. Husband_Udee_IDI_02

#### Feasibility: at community

In qualitative interviews, the HCK custodians reported being comfortable in filling the register for tracking the kit. Only one mentioned having encountered issues in filling the form due to difficulty in understanding the language in the form. Overall, most custodians were happy with their new role. One HCK custodian stated:

I am happy in my work because I want mothers to not face any problems, and I am also a mother, so I am happy to be chosen for this service. If there is anything else, I will do it, and it is going well. I haven't had any problems yet. Custodian_Hidi_KII_03

Some stated that their role as custodian interfered with their daily activity. One custodian raised the challenge she had encountered:

When I am working on something, sometimes it interrupts me and takes time when checking and giving the materials. Custodian_ Denkaka_KII_02

To facilitate availability of the HCKs in their absence, some custodians delegated other family members to carry out their role, thereby reducing the chance of a family not getting the kit. HEWs appreciated the role of HCK custodians as they helped keep the kits safe.

Some of the HEWs stated that collecting the checklist from the custodians was a challenge. One HEW stated:

Whenever we went, we checked all their checklists, materials, what is available or not, who took if their signatures were there, saw how many checklists and for whom they were given, collected, and brought that here. HEW_ Hidi_KII_01

##### Feasibility: at home

In qualitative interviews, women were able to use the checklist and devices and even most of the least educated women reported using the contents of the HCK. A postnatal woman explains how she used the kit:

I am illiterate myself and I am confident I can use the checklist and devices for myself and even for others too because of the advice given at the health centre and I was also able to understand the pictorial display. Mother_Bekejok_IDI_01

However, the checks were more focused on the devices, while other danger signs without the devices were given less attention. Women and family members who had not done the recommended assessments thought that the devices could assess the remaining checklist items. A mother stated the following:

I was sure, because they explained to us that if there is a problem, if there is pain, it is the device that tells you without speaking, if it lights up red, I will say there is a problem. Mother_Katila_IDI_04

For the HEWs who did their home visits, they reported that most of the FPNC families managed to use the kit and checklist properly. HEWs stated that although some families contacted them to solve challenges encountered on how to use the checklist and kit (BP cuff and thermometer), they highlighted that the custodians were normally the first ones to be contacted when families encountered a challenge. HEWs recommended giving more training to empower the HCK custodians as they are closer to the community.

### Acceptability

Acceptability themes included women who have given birth and their families’ self-care experience with the FPNC approach and the acceptability of the FPNC model by healthcare providers and custodians (online supplemental table 3).

#### Self-care experience of women who have given birth and their families

Women’s and families’ acceptance of FPNC was further described through the agency they felt using the self-care approach. The checks for the women who have given birth were done by family members, and the newborn checks were mainly done by their mothers. Women who have given birth reported being pleased with the help they received from their husbands/partners and family members to conduct the health checks at home. The women described what they liked:

When they [family members] started taking my temperature and blood pressure regularly at home, I was especially happy. I appreciate the information that was given to us. Mother_ Giche_IDI_01

I am very happy with my health because I will not make my families worried about my health and I myself will not be worried as well. Because this one [FPNC] is here to help me, this item is not for someone else. I am the one who explains to my husband when it [baby] has a problem or if I am sick. He will help me by bringing and checking using the item and then he will understand whether it is certain or not. He has faith in it, though nothing has happened to me, I am happy with it. Mother_Godino_IDI_02

When people offer their help and support, it is a demonstration of their care and love for you. It’s wonderful to have loved ones who are there for you in times of need. Mother_ Udee_IDI_02

Husbands also were happy and appreciated that they were trusted to follow and actively support the health of their wife/partner and newborn. They reported that with FPNC their engagement was different from previous births when they were not as actively involved, in part due to sociocultural norms. A husband describes how he was more engaged in the postnatal period:

I will take care of my baby before anyone else. As per tradition, males are not allowed to go inside where the mother who delivered is resting, and they never eat what is prepared for the mother. But now, I take care of both the mother and the baby. Husband_Denkaka_IDI_02

Another husband explained:

Previously, with our tradition … there wasn’t much we understood until they got very sick. But now we have understood a little because of this. We have understood that it is very useful to have health follow-up even when the kit is not brought. There are those who never even go, delivering at home. But for us now … the other children were born at home; now I have the awareness that there is follow-up after birth, before birth. The awareness we found after birth gave us freedom, that’s it. Husband_Denkaka_IDI_01

In addition, older children, mothers-in-law and sisters provided checks to mothers and newborns. An eldest son describes what his role was in the FPNC:

We assisted her in maintaining her hygiene, and I have been taking the newborn’s and mother’s blood pressure and temperature. I’ve been filling the checklist. FM_Gichi_IDI_01

#### Healthcare providers’ and managers’ attitudes towards the FPNC approach

Discharge counsellors had a positive attitude towards the FPNC approach, stating it benefited the community, helped women take care of their health and contributed to maternal health-seeking behaviour. A discharge counsellor explained:

…so I strongly support the program. The previous trend is ignoring the family and only focusing on the mother. When there is a danger sign the mother and family incline on the traditional way of treatment. So, in my experience involving the family in the counseling helped to pick postnatal complication easily and helped to seek medical care in the health facilities. DC_Hidi_KII_01

Most HEWs stated that FPNC has made things easier for them because the women learnt to care for themselves with their families’ help. HEWs reported that women, families and communities have an increased awareness to do more health checks, especially BP measurements. Family members and women had contacted them on identifying danger signs. The HEWs stated that when families expressed concerns around identified danger signs, they were appropriate and HEWs managed them accordingly.

Most managers agreed that the FPNC approach has made it possible for many women to assess their health in the postnatal period at home. They also implied that early recognition of postnatal problems and follow-up by HEWs has become easier since the family and women who have given birth were doing the checks daily. A health manager explained why he supported the approach:

Yes, I support [FPNC], because people might get hurt when they hesitate to come to health centre thinking that they might get better after a day or after. Before the FPNC, only the mother used to be counselled but now the whole family gets the counselling, so the attention increased and the equipment [to identify a danger sign] alarms them. HM_Godino_KII_01

### Sustainability of FPNC approach

Sustainability themes considered the health managers’ perspectives, included their commitment to and challenges around the HCK (online supplemental table 4). All health managers confirmed their commitment to sustaining FPNC as they saw the benefits to the women and their communities. They recommended integrating the FPNC approach into monthly and quarterly reports to increase accountability and responsibility for monitoring the approach’s progress. They also mentioned that issues related to the resource supply and kit maintenance and replacement might pose a challenge to FPNC’s sustainability. A health manager described the sustainability of FPNC this way:

It needs attention from higher levels of government, woreda, and community. This needs supplies so it needs higher level involvement. There are procedures for ANC [antenatal care], PNC, and others that are set by the government, so it would be good if this has such procedures from the government. If training is given and the supplies are provided, I believe this can work. HM_Godino_KII_01

### Discussion

This study used a mixed-methods approach to comprehensively evaluate the acceptability, feasibility and sustainability of the FPNC model, drawing on the perspectives of diverse stakeholders to capture a holistic understanding. The FPNC model was implemented across four health centres in the Ada’a District, Oromia Region, Ethiopia. The self-care model demonstrated high feasibility and acceptability, particularly due to the simplicity of the HCK with pictorial and coloured descriptions and accessibility of the materials provided through the HCK custodians located within the community, which were designed for varied literacy levels. At both the facility and community levels, participants reported that the intervention positively influenced families—particularly husbands/partners—by engaging them to support mothers during the postnatal period starting with involvement in postnatal discharge counselling, bringing and returning the HCK, completing the checklist and doing the postnatal assessments. Many women reported increased confidence in recognising danger signs that required urgent medical attention. This aligns with a broader finding that culturally appropriate interventions tend to achieve higher rates of success via self-care mechanisms.^12^ After 6 months, all health centres continued to use the FPNC model, and almost all of the HCKs remained functional and in use.

The FPNC model aimed to address the extremely low coverage of PNC in Ethiopia.^24 25^ The FPNC intervention was effective at significantly increasing PNC coverage. Before the intervention, fewer than 11% of mothers and newborns received a postnatal check on day 3 and day 7 after birth. However, postintervention, this figure rose to over 95%, indicating notable progress in having timely PNC. Both this paper and the one evaluating FPNC’s effectiveness highlighted that very few women received PNC from an HEW, either at home or in a health facility.^15^

The acceptability and feasibility of the FPNC model largely stem from its alignment with cultural norms. In Ethiopia and sub-Saharan Africa, where women who have given birth traditionally remain at home for recovery, the model’s cultural fit increased acceptance.^19 26 27^ In south-western Ethiopia, 75% of women adhered better to PNC when cultural expectations were respected.^12^ Similar interventions in Uganda and Zambia boosted healthcare engagement by up to 20%.^28 29^ The involvement of respected community figures improved health outcomes and raised PNC coverage by 25%–30%.^8 30^ In our study, despite concerns about workload, custodians retained their roles 6 months postintervention, motivated by the value and respect associated with serving mothers.

Our findings indicated that husbands/partners and family members were involved in the postnatal discharge at health centres and checks done at home. Studies show that inviting and involving husbands/partners and family members in maternal and newborn clinics has resulted in higher engagement in the health of the women and newborns.^1314 31^,^33^ Involving husbands/partners and families in discharge counselling might have resulted in higher engagement following the women and newborn health after birth. Future research on self-care models, such as FPNC, may consider exploring possible long-term effects of involving husbands/partners and families on equitable gender and power dynamics in household decision-making around health.

Despite 28% of the women in the study having no formal education and living in rural areas with typically low literacy levels, the women’s capability to engage in self-care using HCK proved highly feasible. This is consistent with other findings that have reported that visual aids and simplified instructions can bridge literacy gaps in low-resource settings.^34^ Some women and families did misunderstand the requirements for completing all necessary checks, assuming that the devices covered everything. These findings underscore the need to ensure that the information provided to families at discharge is clear and that it emphasises the importance of performing all recommended checks appropriately.

Healthcare providers involved in FPNC embraced the self-care-prompting model. By incorporating structured scripts and visual aids to guide postnatal counselling, the model ensured that health professionals could offer standardised, comprehensive advice without overburdening their workloads.^34 35^ A few did mention that counselling with the level of detail that the script and checklist required took more time, particularly for families with no or minimal literacy, as they had to repeatedly explain the procedure.

6 months after the intervention, 100% of health centres involved in the study continued to implement the FPNC model, and nearly all HCKs were still in use. This reflects a strong level of sustainability, which has been linked to community ownership and system integration. A systematic review found that maternal health programmes in sub-Saharan Africa were more likely to be sustainable when communities felt a sense of ownership and when interventions were integrated into existing healthcare systems.^36^ In this case, the FPNC model’s integration into the local healthcare system and its adaptability to community needs facilitated its continued use, suggesting the potential for broader scalability. However, the short 6-month follow-up period, along with challenges such as the lack of national-level reporting and budget for HCK maintenance, limits understanding of long-term sustainability.

The acceptability, feasibility and sustainability of the FPNC approach is promising. Further research of the innovation using a rigorous evaluation design and as well as cost of implementing FPNC at scale would provide valuable information for policy makers in low-resource settings with low PNC coverage.

### Strengths and limitations

This was a small study so the findings might not be generalisable to other settings due to variations in cultural norms, community and health system structures. Because all the women in this study delivered in a facility, we did not assess the acceptability or feasibility of the model for home deliveries, which remain common in Ethiopia, with rates as high as 49%.^37^ Health centre staff who were excluded from the study due to their involvement in the development of the intervention could have discussed the content and quality of the discharge counselling with staff included in the study. However, other aspects of the intervention required specific materials (a discharge checklist and homecare kit) that were not available in other facilities and required dedicated financial inputs, minimising contamination. This study lacked a concurrent control group, indicating that the findings may be influenced by secular trends. However, the relatively short period between baseline and endline reduced the likelihood. Furthermore, self-reported measures can be subject to social desirability bias. Future research should evaluate FPNC in a rigorous study design like observation-based assessments, quasi-experimental and randomised controlled trials in Ethiopia and other settings, as well as feasibility among families with home births. We recognise that cost is a core implementation outcome. However, our study focused on early implementation outcomes (feasibility, acceptability, sustainability); future evaluation could also include costing of the intervention. The credibility of this study was strengthened through systematic coding and iterative analysis, while transferability was supported through clear description of the study setting and participant characteristics, although findings remain context-dependent and may not fully represent all perspectives. Dependability and confirmability were enhanced by documenting analytic decisions and applying the coding framework consistently, though some influence of researcher interpretation is unavoidable in qualitative analysis.

### Conclusions

The FPNC model’s culturally sensitive, low-literacy tools and integration into the healthcare system have demonstrated significant feasibility, acceptability and initial indications of long-term sustainability for a self-care approach to PNC in Ethiopia’s rural, resource-limited settings. These findings align with broader global health findings, showing that tailored, community-centred self-care interventions can significantly enhance PNC birth, even in the most challenging environments.

## Supplementary material

10.1136/bmjph-2025-003548 online supplemental file 1

## Data Availability

Data are available in a public, open access repository.
