# Supplementary material for: Feasibility, acceptability and sustainability of family-led postnatal care model: a multisite mixed study in Ada’a District, Ethiopia
Source: BMJ Public Health. 2026 May 12;4(2):e003548. doi: 10.1136/bmjph-2025-003548 (PMC13182414; doi:10.1136/bmjph-2025-003548)
Supplement: online supplemental file 1 [file bmjph-4-2-s001.pdf]

Supplemental Table 1: ARC ET FPNC IDI/KII Topic Guide

	Theme	Subtheme	Description	Code	Sample quotes
1.	Feasibility of the FPNC model	Feasibility of implementing the FPNC model at the health centre	Midwives' adherence to the discharge script	Adherence to discharge script	*"In the past, the health care provider might have missed important details while providing the service because they lacked an organized checklist form, but now that FPNC has provided the checklist, we follow it, which helps the health care provider to provide discharge-counseling services at the required level."*
			Understanding of the midwives on the role in PNC approach	Role of midwives in FPNC	*"It's excellent. I offer the services I can. Here, we provide a lab investigation, advice, and identification of a danger sign; if there is none, we counsel the mother and send her home after keeping her for 24 hours to check for the sign. We provide counseling upon discharge."*
		Feasibility of implementing the FPNC model at the community	Understanding of the HEW on the role in PNC approach	Role of Health Extension Workers (HEWs) in FPNC	*"The woreda is unable to allocate budgets from its limited budget to sustain the various projects activities that are coming to our woreda. Therefore, it is challenging to replace, repair, and purchase more devices with the woreda's limited budget, so the project donors must consider the sustainability of the project activity"*

			Challenges HEWs faced in collecting the completed checklist from the Home Care Kit custodians	Challenges in collecting the checklist from custodians	*"Whenever we went, we checked all their checklists, materials, what is available or not, who took if their signatures were there, saw how many checklists and for whom they were given, collected, and brought that here."*
			Understanding of the HCK custodian on the role in PNC approach	Role of Home Care Kit (HCK) custodians	*"I make sure the materials are used for the right purpose, I will give the materials and receive after making sure the materials are not damaged."*
			The home care kit availability and accessibility	Access to the home care kit / custodian	*"I brought the HCK. We got it from HCK custodians, a health extension agent.*<br>*There was an issue with the shortage of equipment because two mothers gave birth simultaneously. The problem is discussed among the house heads, who use turn-taking to address it."*
			Interreference of FPNC role in the HCK custodian daily activity	Daily activity interference for HCK custodians	*"When am working on something sometimes it interrupts me and takes time when checking and giving, the materials."*
		Feasibility of implementing the FPNC model at the household levels	Postnatal mother/family members ability and confidence in using the checklist	Use of the checklist	*"No, we had no trouble completing the checklist.*

			Postnatal mother/family members ability and confidence in using the devices	Use of the devices	*"No, both my sister and I used the thermometer devices."*
			Postnatal mother/family members with low literacy ability and confidence in using the checklist	Low literacy use of the checklist	*"I am illiterate myself and I am confident I can use the checklist and devices for myself and even for others too because of the advice given at the health center and I was also able to understand the pictorial display."*
			Postnatal mother/family members with l ow literacy ability and confidence in using the devices	Low literacy use of the devices	*"I am illiterate myself and I am confident I can use the checklist and devices for myself and even for others too because of the advice given at the health center and I was also able to understand the pictorial display."*
			Husbands' and family support in doing the checks	Help with doing the checks	*"My role is to measure the blood pressure and temperature and follow the instructions provided to me. I was shown how to properly use the device to measure the blood pressure and temperature."*<br><br>*"We assisted her in maintaining her hygiene, and I have been taking the newborn's and mother's blood pressure and temperature. I've been filling of the checklist."*

			Helping the mother with other chores	Family involvement	*"I will take care of my baby before anyone else. As per tradition, males are not allowed to go inside where the mother who delivered is resting, and they never eat what is prepared for the mother. But now, I take care of both the mother and the baby."*
2.	Acceptability of the FPNC Mode	Women's self-care experience with FPNC	Experiences of mothers using the FPNC approach and their perceptions of the home-based monitoring and counseling services.	Mothers' happiness; Mothers' preference for the FPNC approach	*"I am very happy with my health because I will not make my families worried about my health and I myself will not be worried as well. Because this one [FPNC] is here to help me, this item is not for someone else. I am the one who explains to my husband when it [baby] has a problem or if I am sick. He will help me by bringing and checking using the item and then he will understand whether it is certain or not. He has faith in it, though nothing has happened to me, I am happy with it."*
		Family involvement and experience with FPNC	Perceptions and experiences of family members participating in discharge counseling and postnatal monitoring.	Preference to have family in discharge counseling; Family interest; Family involvement; Husbands' preference for the FPNC approach	*"Yes, I do want my family to accompany me as I receive the information."* *"All want to be involved in the counseling. Because it is new and not known they are eager to be part of it."* *"We assisted her in maintaining her hygiene, and I have been taking the newborn's and mother's blood pressure and temperature. I've been filling of the checklist."* <br>*"My role is to measure the blood pressure and temperature and follow the instructions provided to me. I was shown how to properly use the device to measure the blood pressure and temperature"* *"FPNC approach helpful in reducing health risks but the old approach does not provide such services."*

		Acceptability of FPNC among healthcare providers	Health providers' perceptions of the usefulness and effectiveness of the FPNC approach in improving postnatal care and early detection of complications	Health providers' support of the FPNC approach	*"…..so I strongly support the program. The previous trend is ignoring the family and only focused on the mother which led the mother to incline on the traditional way. So, in my experience involving the family helped to pick postnatal complication easily and helped to seek medical care."*
		Acceptability among health managers	Perspectives of health managers on the value of the intervention and its contribution to improving maternal and newborn care.	Health managers' support for FPNC	*"Yes, I support, because people might hurt when they hesitate to come to health center thinking that they might get better after a day or after. Before the FPNC only the mother used to be counseled but now the whole family get the counseling so the attention increased and also the equipment alarms them"*
		Acceptability among custodians	Perspectives of custodians on the FPNC model.	HCK custodians' support to FPNC;	*"Am happy that mothers are getting the help even at home. Mothers used to deliver home, bleed but now they deliver at health facilities and get the follow up at home too. When they see red, they go to health facility"*
3.	Sustainability of the FPNC Mode	Health managers' commitment to sustaining the FPNC model	Health managers' perspectives on the importance of integrating and sustaining the FPNC approach within the health system.	Health managers' perception of FPNC sustainability; Health managers' support for the FPNC approach	*"It needs attention from higher levels from government… if training is given and the supplies are provided, I believe this can work."*
		Challenges to sustaining the Home Care Kit (HCK)	Health managers' concerns regarding financial and	Replacement for damaged devices; Limited budget for	*"The woreda is unable to allocate budgets from its limited budget… it is challenging to replace, repair, and purchase more devices."*

			logistical constraints that could affect the long-term sustainability of the intervention.	procurement and maintenance of devices	

Supplemental Questionnaire

Tool 5: ARC_FPNC_Ethiopia Home-based survey tool for postnatal women (Post-intervention)

Locality:	1. Urban<br>2. Semi-urban<br>3. Rural
Geopoint	
Name of study Health center	1. Bekajo<br>2. Dankaka<br>3. Godino<br>4. Hidi
Health post	1. Bekejo HP<br>2. Golbo HP<br>3. Golo-dhertu HP<br>4. Denkaka HP<br>5. Giche Garababo HP<br>6. Gubesaye HP<br>7. Ude HP<br>8. Akako HP<br>9. Godino HP<br>10. Keteba HP<br>11. Kuftu HP<br>12. Yerer silasse HP<br>13. Deko HP<br>14. Hidi HP<br>15. Karfe HP<br>16. Ketila HP<br>17. Tuludimtu HP
Kebele	1. Bekejo Kebele<br>2. Golbo Kebele<br>3. Golo-dhertu Kebele<br>4. Denkaka Kebele<br>5. Giche Garababo Kebele<br>6. Gubesaye Kebele<br>7. Ude Kebele<br>8. Akako Kebele<br>9. Godino Kebele<br>10. Keteba Kebele<br>11. Kuftu Kebele<br>12. Yerer silasse Kebele<br>13. Deko Kebele<br>14. Hidi Kebele<br>15. Karfe Kebele<br>16. Ketila Kebele<br>17. Tuludimtu Kebele
Village/Gote	
Date of delivery: (dd/mm/yyyy) in EC	
Date of Interview: (dd/mm/yyyy) in EC	__ __ / __ __ / __ __ __ __
Data Collector's Name	
Client name	

Client Study ID #		_______________
**NB: please take the picture of the checklist and save on the tablet for data quality check**		
**Section A: PNC for current delivery (to be completed at day 8 by checking the checklist)**		
1.	On which of the following days did the baby get a PNC check for at least one of the components? (Select all that apply)	1. Day 1<br>2. Day 2<br>3. Day 3<br>4. Day 4<br>5. Day 5<br>6. Day 6<br>7. Day 7
2.	On which of the following days did the mother get a PNC check for at least one of the components? (Select all that apply)	1. Day 1<br>2. Day 2<br>3. Day 3<br>4. Day 4<br>5. Day 5<br>6. Day 6<br>7. Day 7
	Number of delivery	
**Section B. Baby Assessment –** Day 1 (Abstract data from checklist)		
3.	Filtering question: If “1” is selected for Q#1	
4.	Feeling well	1. Normal<br>2. Not-normal<br>99 Not checked
5.	Breast feeding	1 Normal<br>2 Not-normal<br>99. Not checked
6.	Burping	1. Normal<br>2. Not-normal<br>99 Not checked
7.	Skin care	1. Normal<br>2. Not-normal<br>99. Not checked
8.	Umbilical cord	1. Normal<br>2. Not-normal<br>99. Not checked
9.	Breathing	1. Normal<br>2. Not-normal<br>99. Not checked
10.	Temperature	1. Normal<br>2. Not-normal

		99. Not checked
11.	Vaccination (Polio 0)	0. No<br>1. Yes<br>99. Not checked
12.	Vaccination (BCG)	0. No<br>1. Yes<br>99. Not checked
**Baby Assessment- Day 2**		
13.	Filtering question: If “2” is selected for Q#1	
14.	Feeling well	1. Normal<br>2. Not-normal<br>99. Not checked
15.	Breast feeding	1. Normal<br>2. Not-normal<br>99. Not checked
16.	Burping	1. Normal<br>2. Not-normal<br>99. Not checked
17.	Skin care	1. Normal<br>2. Not-normal<br>99. Not checked
18.	Umbilical cord	1. Normal<br>2. Not-normal<br>99. Not checked
19.	Breathing	1. Normal<br>2. Not-normal<br>99. Not checked
20.	Temperature	1. Normal<br>2. Not-normal<br>99. Not checked
**Baby Assessment- Day 3**		
21.	Filtering question: If “3” is selected for Q#1	
22.	Feeling well	1. Normal<br>2. Not-normal<br>99. Not checked
23.	Breast feeding	1. Normal<br>2. Not-normal<br>99. Not checked
24.	Burping	1. Normal<br>2. Not-normal<br>99. Not checked

25.	Skin care	1. Normal<br>2. Not-normal<br>99. Not checked
26.	Umbilical cord	1. Normal<br>2. Not-normal<br>99. Not checked
27.	Breathing	1. Normal<br>2. Not-normal<br>99. Not checked
28.	Temperature	1. Normal<br>2. Not-normal<br>99. Not checked
**Baby assessment – Day 4**		
29.	Filtering question: If “4” is selected for Q#1	
30.	Feeling well	1. Normal<br>2. Not-normal<br>99. Not checked
31.	Breast feeding	1. Normal<br>2. Not-normal<br>99. Not checked
32.	Burping	1. Normal<br>2. Not-normal<br>99. Not checked
33.	Skin care	1. Normal<br>2. Not-normal<br>99. Not checked
34.	Umbilical cord	1. Normal<br>2. Not-normal<br>99. Not checked
35.	Breathing	1. Normal<br>2. Not-normal<br>99. Not checked
36.	Temperature	1. Normal<br>2. Not-normal<br>99. Not checked
**Baby assessment – Day 5**		
37.	Filtering question: If “5” is selected for Q#1	
38.	Feeling well	1. Normal<br>2. Not-normal<br>99. Not checked
39.	Breast feeding	1. Normal<br>2. Not-normal<br>99. Not checked

40.	Burping	1. Normal<br>2. Not-normal<br>99. Not checked
41.	Skin care	1. Normal<br>2. Not-normal<br>99. Not checked
42.	Umbilical cord	1. Normal<br>2. Not-normal<br>99. Not checked
43.	Breathing	1. Normal<br>2. Not-normal<br>99. Not checked
44.	Temperature	1. Normal<br>2. Not-normal<br>99. Not checked
**Baby assessment- Day 6**		
45.	Filtering question: If "6" is selected for Q#1	
46.	Feeling well	1. Normal<br>2. Not-normal<br>99. Not checked
47.	Breast feeding	1. Normal<br>2. Not-normal<br>99. Not checked
48.	Burping	1. Normal<br>2. Not-normal<br>99. Not checked
49.	Skin care	1. Normal<br>2. Not-normal<br>99. Not checked
50.	Umbilical cord	1. Normal<br>2. Not-normal<br>99. Not checked
51.	Breathing	1. Normal<br>2. Not-normal<br>99. Not checked
52.	Temperature	1. Normal<br>2. Not-normal<br>99. Not checked
**Baby assessment- Day 7**		
53.	Filtering question: If "7" is selected for Q#1	
54.	Feeling well	1. Normal<br>2. Not-normal<br>99. Not checked

55.	Breast feeding	1. Normal<br>2. Not-normal<br>99. Not checked
56.	Burping	1. Normal<br>2. Not-normal<br>99. Not checked
57.	Skin care	1. Normal<br>2. Not-normal<br>99. Not checked
58.	Umbilical cord	1. Normal<br>2. Not-normal<br>99. Not checked
59.	Breathing	1. Normal<br>2. Not-normal<br>99. Not checked
60.	Temperature	1. Normal<br>2. Not-normal<br>99. Not checked
**Section C- Maternal assessment – Day 1**		
61.	Filtering question: If "1" is selected for Q#2	
62.	How does she feel?	1. Normal<br>2. Not-normal<br>99. Not checked
63.	Headache	1. Normal<br>2. Not-normal<br>99. Not checked
64.	Bleeding	1. Normal<br>2. Not-normal<br>99. Not checked
65.	Breast	1. Normal<br>2. Not-normal<br>99. Not checked
66.	BP	1. Normal<br>2. Not-normal<br>99. Not checked
67.	Temperature	1. Normal<br>2. Not-normal<br>99. Not checked
68.	Swollen face and leg	1. Normal<br>2. Not-normal<br>99. Not checked
**Maternal assessment – Day 2**		
69.	Filtering question: If "2" is selected for Q#2	

70.	How does she feel?	1. Normal<br>2. Not-normal<br>99. Not checked
71.	Headache	1. Normal<br>2. Not-normal<br>99. Not checked
72.	Bleeding	1. Normal<br>2. Not-normal<br>99. Not checked
73.	Breast	1. Normal<br>2. Not-normal<br>99. Not checked
74.	BP	1. Normal<br>2. Not-normal<br>99. Not checked
75.	Temperature	1. Normal<br>2. Not-normal<br>99. Not checked
76.	Swollen face and leg	1. Normal<br>2. Not-normal<br>99. Not checked
**Maternal assessment – Day 3**		
77.	Filtering question: If "3" is selected for Q#2	
78.	How does she feel?	1. Normal<br>2. Not-normal<br>99. Not checked
79.	Headache	1. Normal<br>2. Not-normal<br>99. Not checked
80.	Bleeding	1. Normal<br>2. Not-normal<br>99. Not checked
81.	Breast	1. Normal<br>2. Not-normal<br>99. Not checked
82.	BP	1. Normal<br>2. Not-normal<br>99. Not checked
83.	Temperature	1. Normal<br>2. Not-normal<br>99. Not checked
84.	Swollen face and leg	1. Normal<br>2. Not-normal

		99. Not checked
**Maternal assessment- Day 4**		
85.	Filtering question: If "4" is selected for Q#2	
86.	How does she feel?	1. Normal<br>2. Not-normal<br>99. Not checked
87.	Headache	1. Normal<br>2. Not-normal<br>99. Not checked
88.	Bleeding	1. Normal<br>2. Not-normal<br>99. Not checked
89.	Breast	1. Normal<br>2. Not-normal<br>99. Not checked
90.	BP	1. Normal<br>2. Not-normal<br>99. Not checked
91.	Temperature	1. Normal<br>2. Not-normal<br>99. Not checked
92.	Swollen face and leg	1. Normal<br>2. Not-normal<br>99. Not checked
**Maternal assessment – Day 5**		
93.	Filtering question: If "5" is selected for Q#2	
94.	How does she feel?	1. Normal<br>2. Not-normal<br>99. Not checked
95.	Headache	1. Normal<br>2. Not-normal<br>99. Not checked
96.	Bleeding	1. Normal<br>2. Not-normal<br>99. Not checked
97.	Breast	1. Normal<br>2. Not-normal<br>99. Not checked
98.	BP	1. Normal<br>2. Not-normal<br>99. Not checked
99.	Temperature	1. Normal<br>2. Not-normal

		99. Not checked
100.	Swollen face and leg	1. Normal<br>2. Not-normal<br>99. Not checked
**Maternal assessment- Day 6**		
101.	Filtering question: If “6” is selected for Q#2	
102.	How does she feel?	1. Normal<br>2. Not-normal<br>99. Not checked
103.	Headache	1. Normal<br>2. Not-normal<br>99. Not checked
104.	Bleeding	1. Normal<br>2. Not-normal<br>99. Not checked
105.	Breast	1. Normal<br>2. Not-normal<br>99. Not checked
106.	BP	1. Normal<br>2. Not-normal<br>99. Not checked
107.	Temperature	1. Normal<br>2. Not-normal<br>99. Not checked
108.	Swollen face and leg	1. Normal<br>2. Not-normal<br>99. Not checked
**Maternal assessment – Day 7**		
109.	Filtering question: If “7” is selected for Q#2	
110.	How does she feel?	1. Normal<br>2. Not-normal<br>99. Not checked
111.	Headache	1. Normal<br>2. Not-normal<br>99. Not checked
112.	Bleeding	1. Normal<br>2. Not-normal<br>99. Not checked
113.	Breast	1. Normal<br>2. Not-normal<br>99. Not checked
114.	BP	1. Normal<br>2. Not-normal

		99. Not checked
115.	Temperature	1. Normal<br>2. Not-normal<br>99. Not checked
116.	Swollen face and leg	1. Normal<br>2. Not-normal<br>99. Not checked
**Section D- Care seeking behavior**		
117.	For the abnormality detected on the baby assessment check list **or any other danger sign and symptoms**, did you seek care for the baby?	0. No ------ Skip to 120<br>1. Yes<br>98. DK
118.	For which symptoms did you seek care? (Multiple answers possible)	1. The baby's general wellbeing<br>2. Breastfeeding<br>3. Burping<br>4. Skin care/color<br>5. Umbilical cord<br>6. Breathing pattern<br>7. Body temperature<br>8. Other specify<br>98. DK
119.	Where did you seek care?	1. Health post<br>2. Health center<br>3. Government Hospital<br>4. Private clinic/ private hospital<br>5. Traditional healer<br>6. spiritual healer<br>7. Other specify<br>98. DK
120.	For the abnormality detected on the maternal assessment check list or **any other danger sign and symptoms,** did you seek care for yourself?	0. No ------ Skip to 123<br>1. Yes<br>98. DK
121.	For which of the symptoms did you seek care? (Multiple answer possible)	1. Headache<br>2. Bleeding<br>3. Breast<br>4. BP<br>5. Temperature<br>6. Swollen face and leg<br>7. None (all findings were normal) àSKIP TO 123<br>8. DK
122.	Where did you seek care?	1. Health post

		2. Health center<br>3. Hospital<br>4. Private clinic/ private hospital<br>5. Traditional healer<br>6. spiritual healer<br>7. Other specify<br>98. DK
	Section E- Feasibility	
123.	How did you find the use of BP device for you and your family?	1. We were able to use the BP device without difficulty<br>2. It was difficult for us to use the BP device at the beginning, but we were able to use it with more time<br>3. It was difficult to use the BP device<br>4. We did not use the BP device<br>5. The machine was not functional<br>6. Other specify<br>99. DK
124.	How did you feel using of BP device?	1. I was confident using the BP device<br>2. I was not confident using the BP device<br>3. I did not use the BP device<br>4. Not sure<br>5. Other specify ________________________
125.	How did you find the use of temperature device for you and your family?	1. We were able to use the temperature device without difficulty<br>2. It was difficult for us to use the temperature device at the beginning, but we were able to use it with more time<br>3. It was difficult to use the temperature device<br>4. We did not use the temperature device<br>5. The machine was not functional<br>6. Other specify______________________<br>99. DK

126.	How did you feel using of temperature device?	1. I was confident using the temperature device<br><br>2. I was not confident using the temperature device<br><br>3. I did not use the temperature device<br><br>4. Not sure<br><br>5. Other specify
127.	How did you find the use of checklist for you and your family?	1. We were able to use the checklist without difficulty<br><br>2. It was difficult for us to use the checklist at the beginning, but we were able to use it with more time<br><br>3. It was difficult to use the checklist<br><br>4. We did not use the checklist<br><br>5. Other specify<br><br>99. DK
128.	How did you feel using of the checklist?	1. I was confident using the checklist<br><br>2. I was not confident using the checklist<br><br>3. I did not use the checklist<br><br>4. Not sure<br><br>5. Other<br><br>99. DK
129.	Did you/ your family communicate with a family member used the HCK previously?	0. No<br>1. Yes<br>99. DK
130.	Did you face any challenges in receiving the home care kit from the custodian?	0. No ------- Skip to 132<br>1. Yes<br>99 DK
131.	What were the challenges you faced in receiving the home care kit from the custodian?	1. The HCK custodians were not available at the time we tried to retrieve it

	(Multiple answers are possible)	2. There were no HCKs available at the time we tried to retrieve it<br>3. The designated place was too far from where I live<br>4. We were confused where to retrieve the HCK<br>5. I didn’t have a family member available to retrieve the HCK<br>6. other specify<br>7. 99. DK
132.	What challenges did you face while using the home care kit at home? (Multiple answer possible)	1. The BP apparatus became unfunctional<br>2. The thermometer became unfunctional<br>3. I didn’t have a family member to help me with the HCK<br>4. I didn’t face any challenges<br>5. Other specify<br>99. DK
133.	Did you face any challenges in returning the home care kit to the custodian?	1. No ------- Skip to 135<br>2. Yes<br>99 DK
134.	What were the challenges you faced in returning the home care kit to the custodian?	1. The HCK custodians were not available<br>2. The designated place was far from where I live<br>3. I didn’t have a family member to return the HCK<br>4. other specify<br>99. DK
135.	Did you use the photo health education booklet?	1. No ------- Skip to 138<br>2. Yes<br>99 DK
136.	Did you face any challenges with using the photo booklet?	1. No ------- Skip to 138<br>2. Yes<br>99 DK
137.	If yes, what were the challenges? Write the challenges	-------------------------------------<br>-------------------------------------
	Section F. Acceptability	
138.	Which PNC approach do you prefer?	1. Traditional PNC (PNC by a health care provider)

		2. FPNC<br>3. This is my first delivery and can't compare with traditional PNC & FPNC<br>99. DK
139.	How did you like this approach of monitoring your health and your baby's health at home?	1. I liked it a lot<br>2. I like it<br>3. I was neutral about it<br>4. I did not like it<br>5. I really did not like it<br>6. Not sure/DK
140.	Do you feel confident that you and your baby are receiving good PNC care when doing assessments with the HCK at home?	0. No<br>1. Yes<br>99. DK
141.	How confident are you in the quality of conducting assessments with the HCK at home?	1. Highly confident<br>2. Moderately confident<br>3. Not confident<br>4. Not sure/DK
142.	What type of PNC would you prefer to use after a future delivery?	1. FPNC home-based approach<br>2. PNC by a HEW<br>3. Seek PNC at the health post<br>4. Seek PNC at the health center<br>99. DK
143.	Would you recommend the FPNC approach to other mothers?	0. No<br>1. Yes<br>99. DK
144.	Did your family support you in the FPNC approach (were they involved in the FPNC)?	0. No<br>1. Yes<br>99. DK
145.	Was your family happy to support and help you during the postnatal period using the FPNC approach?	0. No<br>1. Yes<br>99. DK
146.	Which of your family members was the most involved in the FPNC approach?	1. Husband<br>2. Mother<br>3. Sister<br>4. Brother<br>5. In-laws

		6. Child<br>7. Other specify<br>99. DK
147.	Which of your family member's role did you prefer the most in the FPNC approach?	1. Husband<br>2. Mother<br>3. Sister<br>4. Brother<br>5. In-laws<br>6. Child<br>7. Other specify<br>99. DK
148.	Did anyone else in your family use the BP device or thermometer to monitor their own health?	0. No --- Skip to 151<br>1. Yes<br>99. DK
149.	If yes, who else used the devices? (multiple answer possible)	1. Husband<br>2. Mother<br>3. Sister<br>4. Brother<br>5. In-laws<br>6. Child<br>7. Other specify<br>99. DK
150.	Which of the health care devices did they use to monitor their own health?	1. BP device<br>2. Temperature device<br>3. Both<br>99 DK
151.	Which of your family member had difficulties in using the checklist? (multiple answer possible)	1. Husband<br>2. Mother<br>3. Sister<br>4. Brother<br>5. In-laws<br>6. Child<br>7. Other specify<br>99. DK
152.	Which of your family member had difficulties in using the BP device?<br><br>(multiple answer possible)	1. Husband<br>2. Mother<br>3. Sister<br>4. Brother<br>5. In-laws<br>6. Child<br>7. Other specify<br>99. DK

153.	Which of your family member had difficulties in using the thermometer? (multiple answer possible)	1. Husband<br>2. Mother<br>3. Sister<br>4. Brother<br>5. In-laws<br>6. Child<br>7. Other specify<br>99. DK
154.	Which of your family member had difficulties in using the photo booklets?<br><br>(multiple answer possible)	1. Husband<br>2. Mother<br>3. Sister<br>4. Brother<br>5. In-laws<br>6. Child<br>7. Other specify<br>99. DK
155.		
156.	Were you satisfied with your family member support in the FPNC?	0. No<br>1. Yes<br>99. DK
157.	Which PNC approach does your family members prefer?	1. Traditional PNC<br>2. FPNC<br>3. This is my first delivery and can't compare traditional PNC and FPNC<br>99. DK
158.	Do you think your family feels confident in the FPNC approach?	0. No<br>1. Yes<br>99. DK
159.	How involved was your husband/partner in your FPNC care?	0 Very involved ----- Skip to 161<br><br>1 Somewhat involved ---- skip to 161<br><br>2 Not involved
160.	If he was **not** involved: Would you have wanted him to be more involved?	0. No<br>1. Yes<br>99. DK
161.	If he **was** involved:<br><br>How did his involvement feel?	1. welcome/ liked his involvement,<br><br>2. sometimes welcome,<br><br>3. not welcome (e.g., intrusive or pushy)

162.	Did the FPNC kit increase his interest in your recovery and the baby's well-being?	0. No<br>1. Yes<br>2. DK
163.	If this is not your first baby, think back to your previous postpartum experience. Compared to then, how involved is your husband now in: checking on your well-being, sharing household chores, sharing in baby care.	A. Highly involved<br>B. Moderately involved<br>C. Not involved<br>D. Not sure
164.	Compared to before the FPNC intervention, how much do you and your husband/partner discuss important issues together?	1. Discussed frequently<br>2. Discussed Sometimes<br>3. Never discussed<br>4. Not sure
	**Section G - Self-Efficacy/Empowerment**	
165.	Who is the primary decision maker for your health?	A. Myself<br>B. My husband/partner<br>C. Shared decision /Both myself and my husband/partner<br>D. My Parent/Parents<br>E. My Sister<br>F. My Brother<br>G. In laws<br>H. Other specify_________________
166.	Who is the primary decision maker for your baby's health?	A. Myself<br>B. My husband/partner<br>C. Shared decision /Both myself and my husband/partner<br>D. My Parent/Parents<br>E. My Sister<br>F. My Brother<br>G. In laws<br>H. Other specify_________________
167.	Do you get financial support for your health needs?	0. No skip to 169<br>1. Yes
168.	From whom do you get financial support primarily?	A. Myself<br>B. My husband/partner<br>C. Shared decision<br>D. My Parent/Parents<br>E. My Sister

		F. My Brother<br>G. In laws<br>H. Other specify________________
169.	Who makes decision about spending money for your or your baby's health primarily?	A. Myself<br>B. My husband/partner<br>C. Shared decision /Both myself and my husband/partner<br>D. My Parent/Parents<br>E. My Sister<br>F. My Brother<br>G. In laws<br>H. Other specify________________
170.	Do you get social support from people who matter most to you?	0. No skip to 172<br>1. Yes
171.	If Yes for the above question, how strong do you think your social support is from people who matter most to you?	1. Very strong<br>2. Moderately strong<br>3. Weak<br>4. Not sure/DK
172.	How confident are you in negotiating with others like health providers or your spouse for what you might want or need more generally?	1. Highly confident<br>2. Moderately confident<br>3. Not confident<br>4. Not sure/DK
173.	How confident are you in communicating your opinion/stands about your health and your baby's needs with others like health providers, families?	1. Highly confident<br>2. Moderately confident<br>3. Not confident<br>4. Not sure/DK
174.	Do you know how to recognize a problem with yourself during your postnatal period?	0. No<br>1. Yes<br>99 Don't know
175.	Do you know what actions to take if you think there is a problem with yourself during your postnatal period?	0. No<br>1. Yes<br>99 Don't know
176.	Do you know how to recognize a problem with your newborn?	0. No<br>1. Yes<br>99 Don't know
177.	Do you know what actions to take if you think there is a problem with your newborn?	0. No<br>1. Yes<br>99 Don't know
178.	I am good at making decisions related to the health of myself and my family	0. No<br>1. Yes<br>99 Don't know

179.	Do you talk with your husband/partner/family about how to keep yours and your baby's health?	0. No<br>1. Yes<br>99 Don't know
180.	How does your husband/partner help with supporting your health?<br>(Multiple answer	A. Asking how you're feeling<br>B. Listening and showing emotional support<br>C. Preparing food for the family or for you.<br>D. Cleaning or doing other house chores<br>E. Holding/soothing the baby<br>F. Caring for older children while you nurse, nap, or visit with friends<br>G. Bringing you water or snacks while you nurse<br>H. Ensuring that you have access to extra nutritious food to support lactation<br>I. Helping with night feeds/diaper changes to help you get enough sleep<br>J. Being supportive of your decision to go to the health facility if a check is needed<br>K. Paying for health fees<br>L. Arranging and paying for transport if health checks are needed<br>M. Washing baby clothes<br>N. Putting baby to sleep
181.	Do you feel confident in making decision related to your health and your family?	0. No<br>1. Yes<br>99 Don't know
182.	If you do not understand something a provider is telling you, do you tell them and ask them to explain in a different way?	0. No<br>1. Yes<br>99 Don't know
183.	Are there things you can do to help prevent health problems and keep yourself and your baby health?	0. No<br>1. Yes<br>99 Don't know
184.	Do you understand the ways in which the body changes and recovers after having a baby?	0. No Skip to 186<br>1. Yes<br>99 Don't know

185.	If yes, to what degree do you understand how your body recovers after having a baby?	A. Understanding<br>B. Some understanding<br>C. Neutral<br>D. Not much understanding<br>No understanding
**Section H: Demographic and Background Information**		
186.	How old are you?	________ Years<br>98 DK<br>99 NR/RF
187.	What is the highest level of school you attended?	1 None<br>2 Primary<br>3 Secondary<br>4 University<br>5 Quaranic (priest school)<br>6 Other (specify) …………….
188.	What is your religion?	1 Orthodox<br>2 Catholic<br>3 Protestant<br>4 Muslim<br>5 Traditional<br>6 Other__________________
189.	What main language do you normally speak at home (mother tongue)?	Check one only:<br>1. Afan Oromo<br>2. Amharic<br>3. Tigrigna<br>4. Welaita<br>5. Gurage<br>7.Other (specify)…………….
190.	What is your marital status?	1 Never married<br>2 Currently married<br>3 Separated<br>4 Divorced<br>5 Widowed<br>6 Cohabiting<br>99 Refused to answer
191.	What is your occupation, that is, what kind of work do you do? Circle all that apply.	1 Home maker<br>2 Farming<br>3 Teaching<br>4 Business<br>5 Small sales<br>6 Crafts or trades work<br>7 Services<br>8 Health work<br>9 Student

		10 Not employed<br>96 Other (specify)…………………………..<br>99 Refused to answer
192.	How do you usually get to the nearest health facility?	0 Walk<br>1 Public transportation<br>2 Personal transportation<br>3 Other _______
193.	How long does it take you to come to get to the nearest health facility? In minute	[______] in Minute
194.	How many times have you given birth before the current delivery?	_______# If 0 skip to 197<br>99 No Response/RF
195.	For the previous last delivery, did you seek a postnatal care from a health care provider ?	0 No Skip to 197<br>1 Yes
196.	If yes, how many times did you visit a health care provider in the first 6 weeks after birth.?	------------------------------------
**Section I: Equity**		
197.	Does your household have… electricity?	0 No<br>1 Yes
198.	… a radio?	0 No<br>1 Yes
199.	…a television?	0 No<br>1 Yes
200.	… a refrigerator?	0 No<br>1 Yes
201.	… an electric mitad?	0 No<br>1 Yes
202.	… a table?	0 No<br>1 Yes
203.	… a chair?	0 No<br>1 Yes
204.	… a bed with cotton/sponge/spring mattress?	0 No<br>1 Yes
205.	Does any member of this household have a bank account?	0 No<br>1 Yes
206.	What is the main source of drinking water for members of your household?	0 Piped to yard / plot<br>1 Other
207.	What kind of toilet facility do members of your household usually use?	0 Pit latrine without slab / open pit<br>1 No facility / bush / field<br>2 Other
208.	What type of fuel does your household mainly use for cooking?	0 Electricity<br>1 Wood

		2 Other
209.	What is the main material of the floor in your household?	0 Earth / sand<br>1 Other
210.	What is the main material of the exterior walls in your household?	0 Bamboo with mud<br>1 Other
211.	What is the main material of the roof in your household?	0 Metal/corrugated iron<br>1 Other

Please thank the woman and end the interview

Tool 6: ARC_FPNC_Ethiopia Checklist for completeness and functionality of the HCK (HCK registry abstraction tool)

Locality:	1. Urban<br>2. Semi-urban<br>3. Rural
Name of designated place	
Zone	
Kebele	
Name of HCK custodian	
Quantity of kits	
HCK receival date (DD/MM/YY) in EC	__ __ / __ __ / __ __ __ __
Designated Person	
Data Collector's Name	
Date of abstraction	

Note: Please take pictures of the form

Sr. No	Name of user	Received Date (DD, MM,YYYY)	BP Machine functional		Infrared thermometer functional		Print materials					Remark
							Booklet available		Checklist returned			
			Yes	No	Yes	No	Yes	No	Yes	No		

Supplemental Interview guides

Tool 7: ARC_FPNC_Ethiopia Key Informant Interview guide for health center discharge counselors

Locality:	1. Urban<br>2. Semi-urban<br>3. Rural
Name of Health center	1. Bekajo<br>2. Dankaka<br>3. Godino<br>4. Hidi
Health post (write)	
Kebele	
Village/Gote	
Profession of Participant	1. Health officer<br>2. Mid Wife<br>3. Nurse<br>4. Other, specify ________________
For how long have you been working in the maternity unit?	1. Less than 1 year<br>2. Between 1 and 5 years<br>3. More than 5 years<br>4. More than 10 years
Date of Interview: (dd/mm/yy)	__ __ / __ __ / __ __ __ __
Data Collector's Name	
Client Study ID #	________________
Client name	

1. How do you feel about the FPNC approach (if needed, share components of FPNC: the discharge process, checklist, home care kit, HCK custodian, family involvement, the fact that families are monitoring their own health)
   a. What did you like about the approach? Why?
   b. What did you not like about the approach? Why?
   c. How did you feel that health workers, mothers, family and the community support the FPNC approach (including discharge process)? How?
2. What is your opinion about the adequacy of the information, advice, and demonstration you give to the mothers before discharge?
3. How do you think that other health workers feel about the FPNC approach (including the discharge process)?
   a. What do you think they liked about the approach?
   b. What do you think they may not have liked?
   c. How did you feel that health workers, mothers, family and the community support the FPNC approach (including discharge process)? How?
4. How do you think mothers, families, and the community feel about FPNC? What do you think they liked about the approach? What do you think they may not have liked?
5. How have family members been involved in the discharge process? Why were people involved (or not)?
   a. What has this looked like?
   b. What about coming into the room?
   c. How was the level of engagement?
6. How have families responded to the information that they can retrieve a HCK and do assessments at home?
7. How was the process for accessing and completing the FPNC checklist? Did you experience any challenges with using the checklist? If so, can you describe the challenges?
8. Are there factors that have affected the effectiveness of your delivery of PNC discharge information to the mother? If so, please describe.
9. Do you think the FPNC approach is enough for families to screen the danger signs? If no, why? Any recommendations?
10. Do you think FPNC is not feasible for illiterate people? How?
11. What suggestions/recommendation do you have about how to improve the FPNC service delivery in the future?
12. Is there anything else you would like to add?

Thank you for your time!

Tool 8: ARC_FPNC_Ethiopia In-depth interview for postnatal women

Locality:	1. Urban<br>2. Semi-urban<br>3. Rural
Kebele	
Village/Gote	
Date of delivery (dd/mm/yyyy) in EC	__ __ / __ __ / __ __ __ __
Date of Interview: (dd/mm/yyyy) in EC	__ __ / __ __ / __ __ __ __
Data Collector's Name	
Client Study ID #	_______________
Client name	

1. How did you feel having your family be present for the discharge counseling? Do you prefer to have your family with you, or would you have preferred to receive the information without your family next to you?
2. What is your opinion about the adequacy of the information, advise and demonstration of the PNC you received from the health facility upon discharge? Do you think you had enough information? If not, what other information would you liked to have had?
3. How did you feel about doing your PNC assessments at home? What did you most like and what did you not?
4. How confident were you that the self-assessments were able to show you that you were healthy? That your baby was healthy?
5. How confident were you that the self-assessments were able to help you or your family detect any problems with your health? With your baby's health? How easy or difficult was it to complete the checklist? why?
6. Do you think the FPNC approach is feasible for both literate and illiterate people?
7. Did you or your family have any difficulty in using the check list? What type of difficulty?
8. Did you or your family have any difficulty in using the BP device? What type of difficulty?
9. Did you or your family have difficulty in using the Temperature device? What type of difficulty?
10. Compared to before doing FPNC, how has your understanding of your own body changed?

    a. Probe: do you have a better sense of well-being vs. illness? More or less worry about how your body is doing? Do you better understand the changes your body goes through when recovering from birth and when lactating?
11. How has the experience of doing FPNC changed the degree to which you can ask for support from your family—and get it? Please describe.
12. Has the experience of FPNC changed your comfort with discussing body issues with your family or a healthcare provider? Please describe.
13. How has the experience of FPNC changed your view of your health? Please describe.
14. Understanding that welcoming a new baby can itself change our relationships, can you describe ways that having your partner participate in FPNC may have contributed to any changes in your relationship with your partner?
    a. Probes: did it make it easier for her to discuss sensitive issues, did your partner increase his empathy during the postpartum period, did she feel a loss of her privacy, etc?
15. Do you think you are able to identify the postnatal danger signs for yourself and/or your baby? How would you know if you or your baby are in danger? What would you do?
16. What was your husband's role in your postnatal care and in the care of your baby? How involved was he? Was his involvement more or less involved than you hoped?
17. How do you feel about the engagement of family members on FPNC approach? What was their role and how were they involved? Were there some family members who took more responsibility?
18. From which family members do you prefer to give FPNC support? Why? Did you receive that support from those family members?
19. Without the HCK and FPNC, when do you think you might have gone to the health post or health center for PNC? Will you go for additional PNC checks at the health center/health post? If yes, when?
20. In the future, where would you prefer to get your PNC? Through FPNC at home, or with a health provider? Why?
21. In what ways can FPNC be improved?
22. If your partner did not participate in FPNC and you would have liked him to, can you think of any changes to the intervention that might encourage partners to be more involved?
23. Did any of your family members try using any of the equipment like thermometer/BP for their own care?
24. Is there anything else you would like to add?

For women who didn't check all the 7 days

25. What was the reason for not checking all the 7 days in the check list?

26. What would make it easier for you to check your health and your baby's health each day?

For women who didn't seek health care for identified danger sign

27. What was the reason for not seeking care for the danger signs you identified for yourself?

28. What do you recommend for the future to seek health care if you have identified a danger sign for yourself?

29. What was the reason for not seeking care for the danger signs you identified for your baby?

30. What do you recommend for the future to seek health care if you have identified a danger sign for your baby?

Thank you for your time!

Tool 9: ARC_FPNC_Ethiopia In-depth interview for the Family members

Sex	1. Male<br>2. Female
Relation to the family	1. Mother<br>2. Mother in-law<br>3. Sister<br>4. Brother<br>5. Grand Mother<br>6. Grand Father<br>7. Father in-law<br>8. Other, Specify____________________
Where does the family member/s live?	1. Live together<br>2. Live in the neighborhood<br>3. Live outside the neighborhood
Locality:	1. Urban<br>2. Semi-urban<br>3. Rural
Kebele	

Village/Gote	
Date of Interview: (dd/mm/yyyy) in EC	__ __ / __ __ / __ __ __ __

1. Did you participate in the discharge counseling process at the health center? If yes, how did you feel about participating in that process? What was your role in the process?
2. When the nurse/midwife told you about the availability of the HCK, how interested were you in retrieving and using the HCK?
   Probe: If interested, what was it about the HCK that sounded interested? If not interested, why was this the case?
3. After observing the nurse/midwife use the BP device, how confident were you that you would be able to use it at home? Checklist? Thermometer?
   Probe: If not confident, why was this the case?
4. Who in the family retrieved the HCK? If you retrieved it, was it available when you arrived? Did you experience any challenges retrieving it?
5. Where do you recommend the HCK be located in the community?
6. What were your involvement in FPNC approach? For instance, helping to use the BP device, thermometer, and checklist.
7. How does involvement of family members in caregiving help the women during postnatal period?
8. Did you have any difficulty in using the checklist? What type of difficulty?
9. Did you have any difficulty in using the BP device? What type of difficulty?
10. Did you have any difficulty in using the Temperature device? What type of difficulty?
11. How did you provide support for the mother during the postnatal period? Did your support differ with this approach than any support you might have provided for other postnatal women or newborns?
12. How did you provide support for the baby during the postnatal period?
13. Do you think you are able to identify danger signs for a mother and baby? What would you do if you identified danger signs for the mother and/or the baby?
14. How did the mother respond to your participation in FPNC?
15. How did you decide within your family who would help with the FPNC (for example, you assisting with the checks vs her partner? Or did you take turns?)
16. What are your impressions of having the HCK kit in your home so that your family member and baby to be able to monitor their health?
17. Did other members in your family use the BP device or thermometer to measure their own health? If yes, what was their experience?
18. How do you think FPNC compares to other care that you seek at a health post or health center?
19. If the HCK and FPNC had not been available to you in your community, when do you think you might have gone to the health post or health center for PNC?
20. Will you go for additional PNC checks at the health center/health post? If yes, when?

21. Would you recommend that the HCK/FPNC be introduced in other communities? Why or why not?
22. What would you suggest to be improved in FPNC?

Thank you for your time.

Tool 10: ARC_FPNC_Ethiopia In-depth interview for the Husbands/Partners

Locality:	1. Urban<br>2. Semi-urban<br>3. Rural
Kebele	
Village/Gote	
Date of Interview: (dd/mm/yy)	__ __ / __ __ / __ __ __ __

1. Did you participate in the discharge counseling process at the health center? If yes, how did you feel about participating in that process? What was your role?
2. When the nurse/midwife told you about the availability of the HCK, how interested were you in retrieving and using the HCK,BP device and thermometer?
3. After observing the nurse/midwife use the BP device, how confident were you that you would be able to use it at home? Checklist? Thermometer?
4. Who in the family retrieved the HCK? If you retrieved it, was it available when you arrived? Did you experience any challenges retrieving it? Would you have preferred that the HCK be located elsewhere in your community?
5. Did you help her perform the checks or did you perform them? How did the two of you decide who would do what and when?
6. If other family members also helped with FPNC, how did your family decide who would do what, and when?
7. Did you have any difficulty in using the check list? What type of difficulty?
8. Did you have any difficulty in using the BP device? What type of difficulty?
9. Did you have any difficulty in using the Temperature device? What type of difficulty?
10. Did you have any difficulty using the health education booklet? What type of difficulty?
11. Have you or others in the family used any of the equipment's like thermometer/BP for your own care? What was your experience using the equipment?
12. Do you think you are able to identify postnatal danger signs of the newborn? How?

13. Do you think you are able to identify postnatal danger signs of the mother? How?
14. What do you do when you identify danger signs for mother and/or the baby?
15. If you identified any danger signs for the mother or baby, who decided whether and when to go to the health facility?
16. What challenge did you face in escorting your wife to the health facility for Postnatal care
17. What support have you provided to your wife and baby since the birth? Did your support differ with this approach than any support you might have provided for other postnatal women or newborns?
18. Can you tell me some examples of things you learned about caring for the mothers after birth? Can you tell me some examples of things you learned about caring for babies?
19. Understanding that welcoming a new baby can itself change our relationships, how has having your participation in FPNC contributed to any changes in your relationship with your partner?
    a. Probes: did it increase your understanding of what she is going through? Did it strengthen the bond between you? Did it create conflict due to a shift in established roles between you?
20. Likewise, do you think that doing the FPNC checks affected how you bonded with your baby?
21. In many communities, it is a little unusual for husbands to actively support wives during the postpartum phase, and for husbands to participate in baby care. How did it feel for you to be different in that way?
22. Did you communicate with any other husbands who had previously checked out the FPNC box? If yes, Was their support helpful?
23. Did any husbands reach out to you for support after you had returned the box? If yes, What kind of advice or support did you provide them?
24. What can husbands do to support other husbands to become more positively involved in postnatal care?
25. Do you prefer the FPNC approach over traditional PNC? Why?
26. What would you suggest to be improved in FPNC?
27. Is there anything else you would like to add?

Thank you for your time!

Tool 11: ARC_FPNC_Ethiopia Key Informant Interview guide for health extension workers

Locality:	1. Urban<br>2. Semi-urban<br>3. Rural

Name of Health post	
Profession of Participant	
For how long have you been working as a HEW?	5. Less than 1 year<br>6. Between 1 and 5 years<br>7. More than 5 years<br>8. More than 10 years
For how long have you been working in the current catchment area?	
Date of Interview: (dd/mm/yy)	__ __ / __ __ / __ __ __ __
Data Collector's Name	
Participant's name	

1. How do you see the FPNC approach?
   a. Do you think it is a good approach or not?
   b. Why yes or why not?
2. Can you describe your role in the FPNC approach?
3. How has the FPNC approach affected your work? Has it made things easier or more difficult?
4. Have you observed or heard from the family or community about any difficulty in using the checklist?
5. Have you observed or heard from the family or community about any difficulty in using the BP device?
6. Have you observed or heard from the family or community about any difficulty in using the Temperature device?
7. Do you think that FPNC is enough to screen the danger signs? If no, why? Any recommendations?
8. Do you think the FPNC has changed the care seeking behavior of the mother and the family?
   a. Why yes and why not? (give examples)

9. Have you encountered a family member who came seeking for care by identifying a danger sign using the FPNC?
   a. How do you describe the appropriateness of the concerns?
   b. What was done for them?
   c. Can you give examples on the case scenarios that you faced?
10. Have you been able to enter data from the checklists into your monthly reports? If yes, how was that experience? Is there additional information that should be included in the checklists for your monthly report?
11. Have you faced any challenges in collecting the FPNC checklist from the custodians?
12. What do you think are the advantages or drawbacks of the FPNC over the traditional PNC approach? (Give examples)

13. Would you recommend that this FPNC approach be introduced in other health centers/health posts/communities? Why or why not?
14. Please give suggestions/recommendation on how to improve the FPNC service delivery in the future?
    a. Do you think the currently selected custodians are appropriate places to place the HCK with regards to accessibility and feasibility?
    b. How do you see the role of the HCK custodians? What do you think can Improve?
15. Is there anything else you would like to add?

Thank you for your time!

Tool 12: ARC-FPNC_Ethiopa Key Informant Interview guide for Health Care Kit Custodians

Locality:	1. Urban<br>2. Semi-urban<br>3. Rural
Name of HCK Custodian	
Associated Health post/s (write)	
Kebele	
Village/Gote	
Profession of the HCK Custodian	
How long have you lived in this community?	9. Less than 1 year<br>10. Between 1 and 5 years<br>11. More than 5 years<br>12. More than 10 years
Date of Interview: (dd/mm/yyyy) in EC	__ __ / __ __ / __ __ __ __
Data Collector's Name	
Participant's name	

1. What are your impressions of the FPNC approach?
2. Can you describe your role in the FPNC approach? Have you faced any challenges in carrying out the FPNC custodian's role?
3. What procedures do you take while handing over and receiving the HCK from clients?
4. How comfortable were you in filling out the HCK register? Did you face any challenges with filling out the home care kit register? If so, what were they?
   a. Do you have any suggestions on how the register might be improved?
5. How do you describe the load of the FPNC approach on your routine work? (Give examples)
6. What frequent questions or feedback do you receive from the clients (examples of specific challenges faced with the clients)
7. Do families usually return all materials in the kit?
   a. Are all materials usually functional upon return? Which device is more susceptible for malfunctioning and why? How often do the devices malfunction?
8. How do you describe client's handling of the HCK?
   a. Are there particular tools that are usually reported to be challenging to use by the family members / clients)
9. How do you describe your interaction with the HEWs?
   a. Has your interactions with them changed? Do you face any challenges in working with them?
10. Was there a time that you didn't have the HCK when the family member came to retrieve the HCK? if yes, what did you do?
11. Do you think that this location is an appropriate place to keep the HCK for the community?
    a. If yes, why?
    b. If not, why? What other places do you suggest to be more appropriate and why?
12. Did any community members ask to use the HCK for non-postnatal monitoring?
13. What recommendations do you have to improve FPNC in the future?
14. Is there anything that you think you can do to make the FPNC more acceptable and easier to the mother and the family?
15. Is there anything else you would like to add?

Thank you for your time!

Tool 13: ARC-FPNC_Ethiopa Key Informant Interview guide for Health managers

Locality:	1. Urban<br>2. Semi-urban<br>3. Rural
Name of Health center	

Health post (write)	
Kebele	
Village/Gote	
Profession of Participant	1. Health officer<br>2. Nurse<br>3. Midwife<br>4. Other, specify ___________
For how long have you been working in the maternity unit as a manager?	13. Less than 1 year<br>14. Between 1 and 5 years<br>15. More than 5 years<br>16. More than 10 years
Date of Interview: (dd/mm/yy)	__ __ / __ __ / __ __ __ __
Data Collector's Name	
Client Study ID #	_______________
Client name	

1. What are your impressions of the FPNC approach?
2. What changes have you noted in PNC services since your health center began to offer FPNC?
3. In your opinion or experience what is the level of adherence to the discharge counseling script by your staff?
4. Do you think the FPNC checklist and devices are enough for families to detect danger signs? If no, why? Any recommendations?
5. What challenges/barriers has your facility experienced implementing FPNC application?
6. Do you have a plan to buy new "HCK" for obsolete one? Do you have a budget for that?
7. Do you prefer the FPNC approach over the traditional one? How?
8. Do you support the FPNC approach? Why?
9. Do you recommend that your health facility continue the FPNC approach in the future?

10. How sustainable is FPNC approach in your health center??
11. How can the FPNC approach be sustained in other health facilities?
12. Do you have suggestions on how to improve the FPNC service delivery?
13. Is there anything else you would like to add?

Thank you for your time!
